# Heart Rate Variability Based Estimation of Maximal Oxygen Uptake in Athletes Using Supervised Regression Models

**DOI:** 10.3390/s23063251

**Published:** 2023-03-20

**Authors:** Vaishali Balakarthikeyan, Rohan Jais, Sricharan Vijayarangan, Preejith Sreelatha Premkumar, Mohanasankar Sivaprakasam

**Affiliations:** 1 Department of Electrical Engineering, Indian Institute of Technology Madras, Chennai 600036, India; ee22s062@smail.iitm.ac.in (R.J.); sricharanv@htic.iitm.ac.in (S.V.); mohan@ee.iitm.ac.in (M.S.); 2 Healthcare Technology Innovation Centre (HTIC), Chennai 600113, India; preejith@htic.iitm.ac.in

**Keywords:** wearable heart rate monitors, heart rate, heart rate variability, cardiorespiratory fitness, machine learning

## Abstract

Wearable Heart Rate monitors are used in sports to provide physiological insights into athletes’ well-being and performance. Their unobtrusive nature and ability to provide reliable heart rate measurements facilitate the estimation of cardiorespiratory fitness of athletes, as quantified by maximum consumption of oxygen uptake. Previous studies have employed data-driven models which use heart rate information to estimate the cardiorespiratory fitness of athletes. This signifies the physiological relevance of heart rate and heart rate variability for the estimation of maximal oxygen uptake. In this work, the heart rate variability features that were extracted from both exercise and recovery segments were fed to three different Machine Learning models to estimate maximal oxygen uptake of 856 athletes performing Graded Exercise Testing. A total of 101 features from exercise and 30 features from recovery segments were given as input to three feature selection methods to avoid overfitting of the models and to obtain relevant features. This resulted in the increase of model’s accuracy by 5.7% for exercise and 4.3% for recovery. Further, post-modelling analysis was performed to remove the deviant points in two cases, initially in both training and testing and then only in training set, using k-Nearest Neighbour. In the former case, the removal of deviant points led to a reduction of 19.3% and 18.0% in overall estimation error for exercise and recovery, respectively. In the latter case, which mimicked the real-world scenario, the average R value of the models was observed to be 0.72 and 0.70 for exercise and recovery, respectively. From the above experimental approach, the utility of heart rate variability to estimate maximal oxygen uptake of large population of athletes was validated. Additionally, the proposed work contributes to the utility of cardiorespiratory fitness assessment of athletes through wearable heart rate monitors.

## 1. Introduction

Physiological and psychological assessment is required to understand the well-being of athletes and to enhance their competency by improving their health and performance [1]. Wearable Heart Rate (HR) monitors play a major role in tracking their health status and performance through continuous and real-time physiological assessment [2]. One such assessment measure is maximal oxygen uptake ($VO_{2max}$), which is primarily used to represent the functional capacity, well-being, and performance of the individual during sports and exercise. $VO_{2max}$ is measured using traditional devices such as metabolic carts accompanied by gas exchange analysers, along with face mask [3]. The increased load caused by the above accessories makes it laborious for the athletes to perform the testing for long durations. To overcome the inherent challenge of the use of the metabolic cart, a wearable HR monitor [4] can be a suitable solution for $VO_{2max}$ assessment thanks to its unobtrusive nature and reliable measurement.

Initially, mathematical formulas were proposed to assess the value of $VO_{2max}$ [5,6,7].

This was followed by the subjective methods which contained set of questionnaires [8,9,10], namely, Physical Activity Rating (PAR), Perceived Functional Ability (PFA) and Profile Of Mood States (POMS), which were used to assess athletes’ $VO_{2max}$. In subjective methods, the validity of $VO_{2max}$ assessment was majorly dependent on athletes’ unstable and inconsistent responses to the questionnaire which may vary according to their prior experience.

This subjective nature of questionnaire could introduce bias, therefore making it unreliable for $VO_{2max}$ assessment. The objective method consists of both indirect and direct method for assessing the $VO_{2max}$ of the individual. Indirect method includes field tests that estimate a person’s $VO_{2max}$ based on their HR, the distance run covered and their time of trial. The direct method measures an individual’s expired gases to analyze their pulmonary ventilation, inspired oxygen, and their expired carbon dioxide [11] while performing protocols such as Bruce, Astrand–Rhyming and Graded Exercise Testing (GET) [3,11,12,13]. The above protocols are used to assess $VO_{2max}$ directly using mathematical models based on the linear relationship between HR during exercise and its corresponding oxygen utilization.

HR and $VO_{2max}$ have physiological dependence with each other during exercise. HR is directly dependent on $VO_{2max}$, as an increase in $VO_{2max}$ results in an increase in HR, since the increased oxygen demand of the muscles stimulates an increase in HR to ensure that the oxygen is delivered to the muscles. Fairbarn et al. [14] conducted an incremental exercise test to investigate the HR and $VO_{2}$ response to increasing levels of exercise intensity in healthy individuals. Their findings demonstrate that there is a strong relationship between HR and $VO_{2}$ during incremental exercise, and that HR can be used as an indirect measure of $VO_{2max}$ in healthy individuals. In a similar way, studies have shown that Heart Rate Variability (HRV) and $VO_{2max}$ are related in such a way that individuals with higher HRV tend to have higher $VO_{2max}$ values, suggesting that a well-functioning cardiovascular system contributes to better physical fitness. Most of the experimental works suggest that the decrease in HRV can be modeled as a function of exercise intensity, which in turn is measured by $VO_{2max}$. Boutcher et al. [15] conducted a study in which association was observed through the change in cardiac vagal modulation of HR as measured by HRV, with respect to the variation in $VO_{2max}$.

In addition to research related to physiological interpretation, experimental studies establish the association of HR and HRV with $VO_{2max}$ through statistical significance metrics. Habibi et al. [7] examined the linear association between $VO_{2max}$ and HR and validated the association through statistical significance. As a result of association observed between HR and $VO_{2}$ during maximal and submaximal exercise testing, several direct methods were employed to predict $VO_{2max}$ from HR. Balderrama et al. [16] used mathematical models to estimate $VO_{2max}$ from HR using a direct method. Furthermore, the following studies [17,18] established a stronger association between HR and $VO_{2max}$, indicating the fact that HR measurements taken during exercise could be used as predictor variables to estimate $VO_{2max}$.

Additionally, HRV has been found to be a predictor of $VO_{2max}$ in some studies with lower HRV values indicating lower $VO_{2max}$ in individuals. Aggarwala et al. [19] conducted a study that involved 59 archers, HRV measures, namely, Standard Deviation SDNN and Percentage of Number of successive NN Intervals that differ by more than 50 ms (PNNI 50) showed a significant impact on the assessment of $VO_{2max}$. The study [20] developed a mathematical model that contained HRV variables to determine $VO_{2max}$. All the above-mentioned studies, using HRV, investigated its relationship with $VO_{2max}$ on a very small sample size and for a particular age range. Therefore, there is a need to examine this association on a large population with a wide age range distribution. Parallelly, association studies have been conducted with Heart Rate Recovery (HRR) and $VO_{2max}$. The recovery period post-exercise is important for restoring the body to its pre-exercise state, promoting optimal performance and health and reducing the risk of injury and illness. The variation in recovery levels from light, moderate and strenuous exercise is determined by specific metabolic and physiological processes such as $VO_{2max}$, HR, HRV, etc.

The oxygen uptake of an individual remains elevated during the period of recovery to help restore the various metabolic processes to their pre-exercise condition [21]. On this front, the use of HR and HRV parameters during the recovery phase of athletes, to comprehend the characteristics of oxygen uptake, has only been investigated in a small number of studies such as [22,23].

Although there are several studies that already put forth the significance of HR and HRV in $VO_{2max}$ estimation, there prevails an open window to examine the same for larger population with a wide age range distribution. In doing so, a robust and reliable Machine Learning (ML) model could be developed and its feasibility could be established in monitoring the performance of athletes through wearable HR monitors. In light of the previous studies and in consideration of their findings, this work proposes the utility of HRV derived from exercise and recovery phase, of a large number of athletes with a wide age range distribution, to assess $VO_{2max}$ by employing a ML approach.

## 2. Related Works

In this section, we consider the research works with perspectives to launch various methodological approach carried out in the form of maximal exercise testing protocol and data-driven models employed to understand the relationship of HR & HRV with $VO_{2max}$. Additionally, to produce relevant features to the data driven models, we provide descriptions of significant feature selection methods.

### 2.1. Maximal Exercise Testing

The classical method of estimating $VO_{2max}$ is during the incremental exercise test [3,24,25,26,27]. These studies focus on estimating the $VO_{2max}$ during the exercise intervention to investigate the long-term changes and monitor the performance and fitness of the individual. The preliminary Cardio Pulmonary Exercise Testing (CPET) was performed using the mechanical devices such as computerized metabolic cart, motorized treadmill, ergometer accompanied with breath-by-breath gas exchange analyzers for measurement of $VO_{2max}$. The oxygen consumption variables are drawn from the face mask connection that was sampled with the analyzer. George et al. [28] involved 100 participants (50 females and 50 males) within the age range of 18–65 years to perform maximal exercise testing. Then, a linear regression model was used with independent variables such as gender, body mass index, treadmill speed and treadmill grade, which resulted in accuracy given by $R^{2}$ value 0.88 and Standard Error Estimate (SEE) of 3.18.

### 2.2. $VO_{2max}$ Estimation Using HR

With improvement in wearable technology, sophisticated algorithms and techniques, effective and reliable data from the athletes are obtained that help in estimating their $VO_{2max}$ through standardized protocols, without affecting the functional capability of the user during the assessment. Neilson et al. [29] chose 105 participants (53 males, 52 females) and calculated the Pearson correlation coefficient between measured and predicted $VO_{2max}$. Multiple linear regression analysis was performed with independent variables such as gender, body mass, PFA, work rate and steady-state HR and the $R^{2}$ value obtained was 0.82, with SEE of 3.36. Brabandere et al. [25] conducted a maximal incremental test on a treadmill which included 31 recreational runners (15 men and 16 women) aged 19–26 years. Ariza et al. [20] included a total of 20 volunteer subjects (10 men and 10 women) with a mean age of 19.8 ± 1.77 years in the study. In the above studies, data-driven models were developed which used both metadata and HR as predictor variables to directly estimate $VO_{2max}$. Certainly, HR features used as predictor variables in the models had a larger effect on accuracy of the $VO_{2max}$ prediction. Although developmental model studies on exercise interventions have been reported to a greater extent, there are only a handful of studies that deal with the recovery window. Suminar et al. [23] proposed an improvement of HR recovery after giving treatment for eight weeks with 24 sessions. The average value of pre $VO_{2max}$ was 30.73 mL/kg/min with a pulse recovery of 100.7 dt/min.

After the training phase, the $VO_{2max}$ improved to 32.82 mL/kg/min with a decreased level of pulse recovery rate to 94.7 dt/min.

### 2.3. $VO_{2max}$ Estimation Using HRV

Ariza et al. [20] calculated the correlation coefficient between $VO_{2max}$ and each of the variables, including body composition, age and HRV, using multivariate regression analysis. The results show that both age and HRV of the participants influenced the final prediction model of $VO_{2max}$, with R = 0.423 in men and R = 0.212 in women [30]. The performance and prediction accuracy of such various regression models were compared with each other to identify the best ML and statistical methods. The survey results showed that SVR-based models performed better than other regression methods to predict $VO_{2max}$. Aggarwala et al. [19] observed that both time domain HRV measure SDNN and PNNI 50 were positively correlated with $VO_{2max}$ with the coefficient values 0.72 and 0.70, respectively.

A cross sectional study [31] investigated the association of HR, HRV with $VO_{2max}$ using stepwise multiple regression analysis. The results indicated that HR, RR interval and PNNI 50 showed significant correlations with $VO_{2max}$.

### 2.4. Correlation-Based Feature Selection

Correlation is a combination of a wrapper-based and a filter-based feature selection [32] method. Initially, the features are ranked based on their Spearman’s correlation coefficient with the dependent variable. Then, the multi-collinearity among the predictor variables is eliminated by removing the feature that has low correlation with the dependent variable and a high correlation with the higher-ranked feature. By performing this process iteratively, the original set of features is condensed into a smaller bunch with less bivariate collinearity. Kumari et al. [33] employed correlation-based feature selection to identify people with alcoholic disorder using recorded brain activity signals. Mitra et al. [34] conducted a correlation-based feature selection study to investigate and classify different types of arrhythmia from ECG signals.

### 2.5. Mutual Information Based Feature Selection

The Mutual Information-based approach [35] employs the principles of filter-based and wrapper-based feature selection methods, similar to the correlation-based approach. The difference lies only in the ranking algorithm where instead of ranking the features based on the correlation coefficient, the features are ranked using their mutual information with the dependent variable. Mian et al. [36] utilized mutual information-based feature selection to classify cardiac arrhythmia similar to the correlation-based approach. Sharma et al. [37] proposed a study that demonstrated the utility of mutual information-based feature selection to distinguish between fatty and normal liver from ultrasound images.

### 2.6. Greedy-Based Feature Selection

Greedy forward and backward feature selection is a conventional solution to the feature selection problem. This method aims to optimize the desired performance metric at each step of the process. To account for the limitation of forward feature selection and backward feature elimination, both are performed recursively until there is no improvement in the selected performance metric. The forward feature selection starts with an empty set, then the first feature is selected such that when a regression model is trained using this feature, it gives the minimum value of the chosen metric. For every iteration, the model performance is optimized such that the combination of new feature and the best features from the last iteration gives the minimum value of the chosen metric.

This process is followed iteratively until there is no further improvement in the model performance by the addition of features. Although the set of features obtained through forward addition is found by optimizing the performance metric at each step, it does not guarantee to be the best possible combination. Therefore, backward feature elimination is performed.

**Figure 1:**
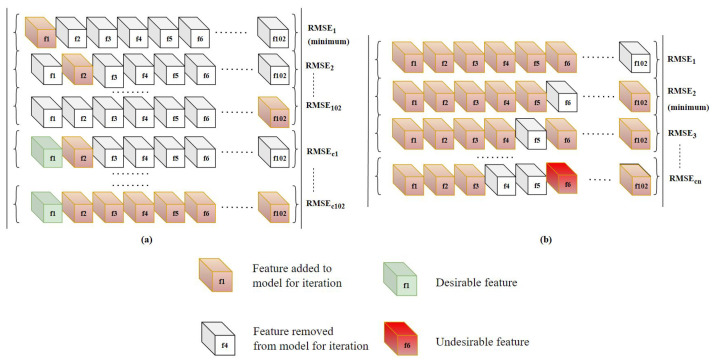
Diagrammatic representation of Greedy-based feature selection. Each sub-figure shows the process of: (**a**) Sequential forward feature selection. (**b**) Sequential backward feature elimination.

In backward feature elimination, a feature is removed, and the regression model is trained using the remaining features. The feature whose removal resulted in a better performance metric is removed from the feature set. Similar to the forward algorithm, this process is recursively performed until there is no improvement in the performance metric. The algorithm finishes when neither the addition of a new feature nor the removal of an existing feature improves the model performance. A single iteration process of greedy forward and backward is represented in the Figure 1. Rodrigues et al. [38] has proposed a automatic segmentation and labeling approach of multimodal biosignal using Self-Similarity Matrix computed with the signals’ feature-based representation. Hui Liu et al. [39], in his work, used features derived from biosignals for Human Activity Recognition using greedy feature selection. Hatamikia et al. [40] used a greedy forward feature selection approach to build emotion recognition system using EEG signals. Mar et al. [41] applied a sequential forward selection approach to assess the quality of ECG in arrhythmia classification. This study [42] implemented greedy forward approach as a feature selector to discriminate 20 different atrial flutter mechanisms using ECG signals.

Through extensive literature study, we observe that data-driven models trained using HR and HRV are reliable for $VO_{2max}$ estimation. Additionally, we infer that the above-mentioned feature selection methods, chosen for this study, have their widespread utility in the biosignal domain for improving the performance of data-driven models.

## 3. Dataset

In this section, we provide details of the data, collected during the incremental protocol, which are used in this study to estimate $VO_{2max}$. Further, we describe the preprocessing steps used to clean the data followed by extraction of features which is given as input to the model.

### 3.1. Dataset Description

The database contained cardiorespiratory measurements obtained from 856 professional athletes of age range (10–63 years old), at Exercise Physiology and Human Performance Lab of the University of Malaga [22,43,44]. The data were collected during GET protocol, a type of exercise test that involves increasing the intensity of the exercise in a controlled manner, usually in incremental step or ramp, to assess the individual’s cardiovascular and respiratory responses to exercise. The athletes performed the test on a PowerJog J series treadmill and the data were collected using CPX MedGraphics gas analyzer system and 12 lead ECG Mortara system.

The gas analyzer measured breath-by-breath respiratory parameters mainly $VO_{2max}$ and a 12-lead ECG Mortara system recorded breath-by-breath HR. The metadata file used for the analysis contained participants’ details limited to age, height and weight.

### 3.2. Data Pre-Processing

Breath-by-Breath HR recorded by the ECG monitor (ECG Mortara) during the test was subjected to pre-processing steps, namely, artifact correction and ectopic beats removal. HR corresponding to RR intervals that were exclusive to range 300–2000 ms was considered as outliers and the ectopic beats were determined using the Karlsson method [45]. Both outliers and ectopic beats identified in the previous step were replaced by values using linear interpolation. Pre-processed HR segments corresponding to exercise and recovery were extracted. Then, HRV features were computed on those segments using time domain, frequency domain and non-linear analysis. Since the monitor was switched off after 3 min of exercising, the HR segments corresponding to recovery phase were inadequate to compute frequency domain HRV as per recommendations [46]. Therefore, HRV during recovery phase was extracted using time and non-linear domain analysis.

In addition, three criteria were taken into account in order to remove unreliable HR segments from both exercise and recovery data. They were as follows:

1. HR and $VO_{2max}$ trends were eliminated if they were out of phase;
2. Consecutive HR that differed by more than 30 bpm between were removed;
3. Data segments which had less than 5 min of HR and $VO_{2max}$ values were excluded;
4. The participant data which had missing HR and $VO_{2max}$ values were removed.

On applying the above criteria, 75 exercise data segments and 20 recovery data segments were removed.

HRV measures obtained from time domain were Root Mean Square of the Differences between Successive RR intervals (RMSSD), Percentage of Number of successive RR Intervals that differ by more than 20 ms (PNNI 20), PNNI 50, Number of successive RR Intervals that differ by more than 20 ms (NNI 20), Number of successive RR Intervals that differ by more than 50 ms (NNI 50) and difference between maximum NNI and minimum NNI (Range NNI) [46]. The primary HRV features from frequency domain analysis [46] were, namely, total power density spectral was obtained with power parameters in Very Low Frequency (VLF) of range 0–0.04 Hz, low frequency (LF) ranging between 0.04–0.15 Hz and high frequency (HF) from 0.15 to 0.4 Hz. For non-linear domain analysis [46], Poincare plot was used to establish short range (SD1) and long-range (SD2) variations in the HR values. Detrended Fluctuation Analysis (DFA) was used as one of the HRV features, due to its potential power to correlate between successive RR intervals over different time scales as expressed by α1 (4–16 beats) and α2 (16–64 beats). Hence, HRV features extracted were further used to estimate $VO_{2max}$.

### 3.3. Feature Extraction

From exercise HR segments, time domain HRV features, namely, ‘NNI 20’, ‘NNI 50’, ‘Range NNI’ along with slope of those features were given as set of input features to the models. Additionally, the frequency domain HRV features ‘VLF’, ‘LF’, ‘HF’ and also slope of the same were also included. Further non-linear domain HRV features, namely, DFA estimates (α1), Poincare plot estimates (‘SD1’ and ‘SD2’) and slope of the same were added to the list. The slope of HRV features were computed to comprehend the characteristic change in HRV over time. Then, time-based features, namely, ‘SlopeSp25’ (slope of HR and time segments corresponding to 0–25% maximum speed), ‘RT25’ (correlation between speed and HR segments corresponding to 0–25% total time), ’RSp25’ (correlation between speed and HR segments corresponding to 0–25% maximum speed), ‘TimeSp25’ (time at which they reached 25% of max speed), ‘TimeHR25’ (time at which their HR was 25% max HR), ‘DurationHR60’ (total time spent in zone 50–60% of max HR) and similar such features were also included. This yielded 101 features associated with exercise data.

The detailed description of each feature derived from HR during exercise is listed in Appendix A. In case of recovery, along with metadata, time domain HRV, slope of time domain HRV, non-linear domain HRV, slope of non-linear domain HRV measures were computed, which yielded 30 features. The distribution of the derived features were tested for normality using Shapiro–Wilk test and observed to be not normally distributed. The derived features from both exercise and recovery HR segments were fed to the model for $VO_{2max}$ estimation.

## 4. Methodology

In order to estimate $VO_{2max}$ using derived HRV features, three machine learning models, namely, Multiple Linear Regression (MLR), Random Forest Regression (RF) and Support Vector Regression (SVR), were chosen in this study. A brief mathematical overview of the working principle of each model and their evaluation approach represented by different metrics are described in this section.

### 4.1. Multiple Linear Regression

MLR is a statistical method used to model a linear relationship between the dependent variable and multiple independent variables. This method aims to fit the linear predictor function as described in Equation (1).

(1) $$y=b_{0}+\sum_{i=1}^{n}b_{i}*x_{i}$$

where *y* is the dependent variable, *n* is the number of predictor variables, $x_{i}$ is the *i*th independent features, also known as a predictor variable, $b_{0}$ is the intercept term and $b_{i}$ is the regression coefficient of the corresponding predictor variable.

The intercept term and the regression coefficients are determined by choosing an appropriate loss function and minimizing it using the gradient descent algorithm. Conventional loss functions are Mean Squared Error (MSE) and Mean Absolute Error (MAE). For this study, the loss function was chosen as MSE because of its convexity and differentiability.

(2) $$MSE=\frac{1}{k}\sum_{i=1}^{k}{\left(y_{i}-\hat{y_{i}}\right)}^{2}$$

where *k* is total data points in the training set, $y_{i}$ is the *i*th data point of the dependent variable and, $\hat{y_{i}}$ is the corresponding prediction.

### 4.2. Random Forest Regression

The decision tree, an integral component of RF, is a non-parametric supervised learning algorithm that creates a tree-like structure to segregate the training data points. The tree comprises a root node, internal nodes, branches and leaves. To find the root node, the dataset is split into two halves and the Sum of Squared Residual (SSR), as described in Equation (3) is calculated with respect to the aggregates of each branch. Then, the threshold of the root node is decided such that after splitting the training data into two branches, it minimizes the SSR.

(3) $$SSR=\sum_{i=1}^{N_{1}}{\left(y_{i}-y_{1}\right)}^{2}+\sum_{j=1}^{N_{2}}{\left(y_{j}-y_{2}\right)}^{2}$$

where $y_{i}$ and $y_{j}$ are the data points in the left and right branches, $N_{1}$ and $N_{2}$ are the number of data points in the left and right branches, and $y_{1}$ and $y_{2}$ are the aggregates of target variables of the left and right branches, respectively.

Similarly, the threshold of internal node is decided such that only the maximum allowable number of data points remains in each node, known as leaf nodes. Even though decision trees have low bias, it tends to overfit because of high variance.

This issue is somewhat rectified by using RF, an ensemble learning algorithm that uses a multitude of decision trees and aggregates their output to give the final prediction. The method that RF employs is called bootstrapping, where it creates multiple decision trees with a subset of features and training set, which slightly increases the bias in the model but improves the performance greatly.

RF regressor has three main hyperparameters which are ‘max. depth’, which signifies the maximum depth of the decision trees, ‘n estimators’, which determines the number of trees in the model, and ‘min. samples split’, which determines the minimum number of samples required to split the internal node.

### 4.3. Support Vector Regression

SVR is a supervised machine learning algorithm that employs the principle of support vector machine to perform a regression analysis. The objective of this approach is to predict the hyperplane and ϵ-insensitive margins for the said hyperplane. The ϵ is a hyperparameter that defines the width of the tube around the estimated function, such that the optimization function does not penalize the data points lying inside the tube. Another important aspect of SVR is that it emphasizes the flatness of the estimated function by minimizing the L2-norm of the coefficient vector. Conventionally, SVR is modeled to estimate a linear function, but the dataset may not adhere to this constraint. Hence, the kernel function is used to project the data into the higher dimensional space and account for the non-linearity. In this study, the Gaussian Radial Basis Function (Gaussian-RBF) was chosen as the kernel because of its improved performance on the dataset. The predictor function, f(x) that needs to be estimated is described in Equation (4).

(4) $$\begin{array}{c} f\left(x\right)=\sum_{i=1}^{N}\left(a_{i}-a_{i}^{*}\right)G\left(x_{i},x\right)+b \end{array}$$

(5) $$\begin{array}{c} G\left(x_{i},x\right)=e^{-\gamma ||x_{i}-x{\left|\right|}^{2}} \end{array}$$

where *x* is the input vector, *b* is the intercept, *G* is the kernel function, *N* is the total number of data points, $x_{i}$ is the $i^{th}$ feature vector whose corresponding observed dependent variable is $y_{i}$, and $a_{i}$ and $a_{i}^{*}$ are the non-negative multipliers used to construct the Lagrangian function from the primal to the dual function.

To solve for the unknowns of the given equation a loss function, L(α) described in Equation (6), needs to be minimized considering the constraints. Due to the possibility that there exists no such solution as a slack term, *C* is added to the convex optimization constraint, which is described in Equation (7).

(6) $$L\left(\alpha \right)=\frac{1}{2}\sum_{i=1}^{N}\sum_{j=1}^{N}\left(\alpha_{i}-\alpha_{i}^{*}\right)\left(\alpha_{j}-\alpha_{j}^{*}\right)G\left(x_{i},x_{j}\right)+\epsilon \sum_{i=1}^{N}\left(\alpha_{i}+\alpha_{i}^{*}\right)-\sum_{i=1}^{N}y_{i}\left(\alpha_{i}-\alpha_{i}^{*}\right)$$

(7) $$\begin{array}{c} \sum_{i=1}^{N}\left(\alpha_{i}-\alpha_{i}^{*}\right)=0\\ i:0\leq \alpha_{i}\leq C\\ i:0\leq \alpha_{i}^{*}\leq C \end{array}$$

As described above, ϵ, γ and C are the hyperparameters that need to be tuned to give an optimal performance which is achieved by an exhaustive search of the parameter space. In this study, this was done using the grid search algorithm along with 5-fold cross-validation.

### 4.4. k-Nearest Neighbor

Outliers are those observations in a dataset that differ significantly from other data points. These outliers can arise because of factors such as erroneous measurements, abnormal physiology and inconsistent data entry.

The existence of these outliers in data skews the data distribution and compromises the generalizability of the prediction models. Therefore, to address this issue, the outliers that defied the internal structure of the data were detected and removed using the k-Nearest Neighbor (k-NN) algorithm proposed by Sridhar et al. [47]

The k-NN algorithm is a non-parametric supervised learning algorithm that has several use cases, including but not limited to outlier detection. The underlying assumption of this method is that, in the multidimensional feature space, similar observations exist in proximity. The proximity is defined based on the distance between each data point and its k nearest neighbors. This local neighborhood is found based on various distance measures, such as Euclidean distance, Manhattan distance or Cosine similarity. Considering the low dimensionality of the feature space, Euclidean distance measure is preferred, also known as the L2 norm of resultant vector, as described in Equation (8).

(8) $$d={\left(x_{a}-x_{b}\right)}^{T}\left(x_{a}-x_{b}\right)$$

where d is the Euclidean distance between vectors, $x_{a}$ and $x_{b}$ in the feature space.

To differentiate between outliers and non-outliers, the aggregate distance of each point with its k nearest neighbors is found and based on a predefined threshold, the distinction is drawn.

### 4.5. Evaluation Metrics

The conventional metrics used to evaluate the performance of predictive regression models are Root Mean Squared Error (RMSE), Mean Absolute Error (MAE), Mean Absolute Error Percentage (MAPE) and Pearson correlation coefficient (R). Out of these, the preferred error metric for the study is RMSE due to its ability to account for the large errors that are highly undesirable [48,49,50]. To provide an linear estimate about the prediction accuracy of $VO_{2max}$ values, R metric is preferred in this study [51,52,53].

## 5. Experimental Approach

This section describes the process of the feature selection methods that is incorporated with the above ML-based approaches to avoid overfitting of the regression models. Additionally, the performance of these models could be improved by providing relevant features. Since there were 101 features for exercise and 30 features for recovery, the feature selection was used to minimize the redundancy of the features, and maximize their relevancy. Therefore, correlation-based feature selection, mutual-information-based feature selection, and a combination of greedy forward and backward feature selection were employed in this study.

### 5.1. Correlation

As mentioned in Section 2.4, correlation-based feature selection allocates the importance to the features based on their absolute value of Spearman’s correlation coefficient with $VO_{2max}$. Since the majority of feature data points were not normally distributed and contained outliers, Spearman’s rank correlation was used to measure the correlation between the features. With respect to exercise, all the 101 independent features were ranked from highest to lowest based on their correlation coefficient. Any feature whose absolute value of correlation with other feature was greater than 0.7 was categorized as highly correlated. The methodology utilized to derive the threshold was based on the empirical observation that the degree of bivariate collinearity within the 90% subset of feature combinations was below 0.7, which is a commonly accepted threshold for assessing multicollinearity in statistical analyses. To avoid the effect of multicollinearity, the features which had lower correlation with $VO_{2max}$ and high correlation with higher-ranked features were pruned. As a result of pruning, the original set of 101 features was reduced to 42 features. Similarly, the above process was performed on 30 such features which was extracted from the recovery window. This resulted in a pruned set of 14 features.

**Figure 2:**
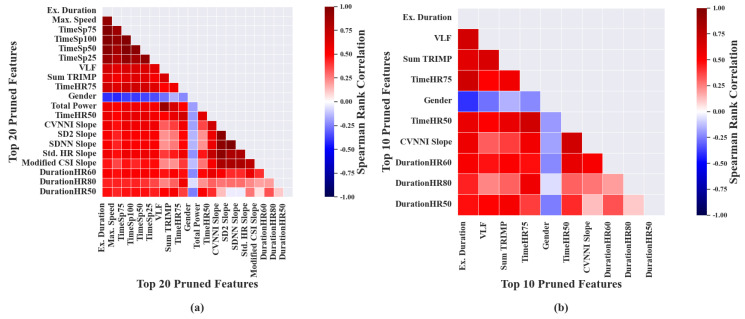
Heat maps depicting bivariate collinearity among predictor variables. Each of the subfigures shows the Spearman’s correlation coefficient between: (**a**) the top 16 ranked features associated with exercise data; (**b**) the top 10 pruned features associated with exercise data.

For the purpose of representation, correlation-based feature selection was performed on 20 features, extracted from exercise segments, which had the highest correlation with $VO_{2max}$. It was noted that the features, namely, ‘Max. speed’, ‘TimeSp25’, ‘TimeSp50’, ‘TimeSp75’ and ‘TimeSp100’ had high correlation with ‘Ex. Duration’ and hence were removed during the pruning phase. Similarly ’Total Power’ was also removed due to its high correlation with ‘VLF’, as shown in Figure 2a. It was observed that the overall bivariate collinearity among the pruned features, as represented in Figure 2b, was relatively lower when compared with the original ranked features. The order in which the listed pruned features, associated with exercise segments, mentioned in Table A1 of the Appendix A are based on category and not in ranked order.

**Figure 3:**
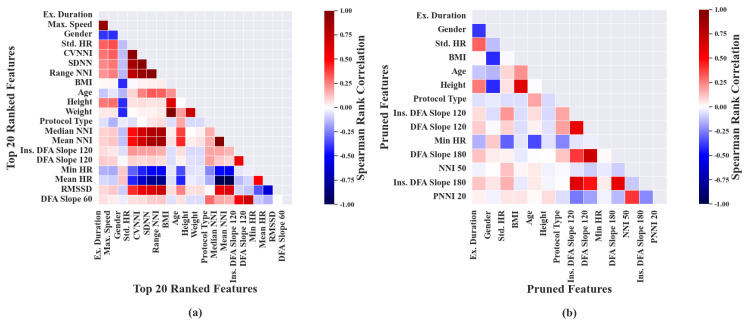
Heat maps depicting bivariate collinearity among predictor variables. Each of the subfigures shows the Spearman’s correlation coefficient between: (**a**) the top 20 ranked features associated with recovery data; (**b**) the pruned features associated with recovery data.

Similarly, the above feature selection was performed on 20 features derived from recovery data that had highest correlation with $VO_{2max}$. As shown in Figure 3a, ‘CVNNI’ and ‘SDNN’ were pruned due to their higher correlation with ‘Weight’, which in turn was removed due to its high correlation with the higher-ranked feature, ‘BMI’. ‘CVNNI’ showed high correlation with ‘Std. HR’ and hence was removed during the pruning phase. The above process was performed iteratively which resulted in 14 pruned features, as shown in Table 1.

Finally, it was observed that the bivariate collinearity among the pruned features associated with recovery, as shown in Figure 3b, was relatively lower when compared with the original ranked features. The pruned features in Table 1 are listed based on category and not in ranked order.

### 5.2. Mutual Information

This approach ranked 101 features extracted from exercise segments based on their mutual information with $VO_{2max}$. The ranked features were subjected to pruning process similar to the one performed during correlation-based feature selection.

As a result of pruning, the original set of ranked features was condensed to 41 features. Similarly, the above process was performed on 30 such features, which were extracted from recovery window.

**Figure 4:**
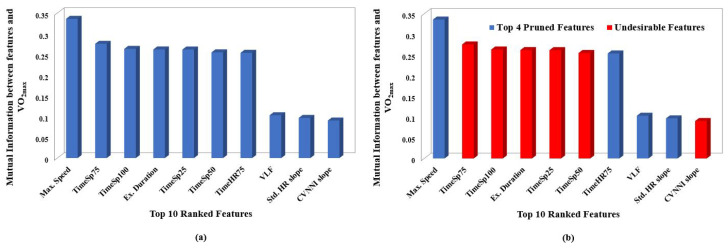
Plots depicting ranking and pruning performed based on mutual information. Each sub plot shows: (**a**) Mutual information between $VO_{2max}$ and top 10 ranked features associated with exercise data. (**b**) Undesirable features removed based on Spearman’s correlation coefficient of exercise data.

For the purpose of representation, Mutual Information-based feature selection was performed on the top 10 ranked features, extracted from exercise segments as shown in Figure 4a. It was noted that the features ‘TimeSp50’, ‘TimeSp75’, ‘TimeSp100’ and ‘Ex. Duration’ had high correlation with ’Max. Speed’ and hence were considered as undesirable features during the pruning phase, as shown in Figure 4b.

Similarly, ‘CVNNI slope’ was also considered undesirable due to its high correlation with ‘Std. HR’. Eventually, the top 10 features were condensed to four features after the pruning process. The order in which the listed pruned features, associated with exercise segments, are mentioned in Table A2 of Appendix A is based upon category and not in ranked order.

**Figure 5:**
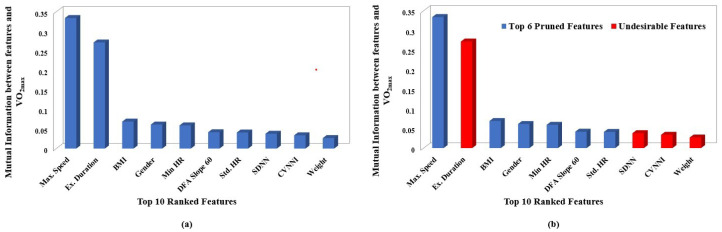
Plots depicting ranking and pruning performed based on mutual information. Each sub plot shows: (**a**) Mutual information between $VO_{2max}$ and top 10 ranked features associated with recovery data. (**b**) Undesirable features removed based on Spearman’s correlation coefficient of recovery data.

Similarly, Mutual Information-based feature selection was performed and the top 10 features, as in Figure 5a, derived from recovery segments, were ranked from highest to lowest. After ordering the features based on Mutual Information, the ranked features were subjected to pruning. The second-highest-ranked feature ‘Ex. Duration’ was removed because of its higher correlation with first ordered feature ‘Max. Speed’. Additionally, lower-ranked features ‘SDNN’, ‘CVNNI’ and ‘Weight’ showed high correlation with their higher-order-ranked features and hence were considered as Undesired Features, as shown in Figure 5b.

The above process was performed iteratively on the original set of ranked features which resulted in 13 pruned features, as shown in Table 2. The pruned features in Table 2 are listed based on category and not in ranked order.

### 5.3. Greedy Forward–Backward Method

The greedy-based sequential feature selection was performed as described in Section 2.6. With respect to the 101 features corresponding to the exercise data and SVR model, the forward and backward algorithm was used iteratively to find the best set of features. It was found that the forward feature selection process yielded 30 features primarily ‘Ex. Duration’, ‘Protocol Type’, ‘BMI’, ‘Gender’, ‘TimeSp50’ etc. Whereas, during the backward elimination of features, three features were removed, namely, ‘PNNI 20’, ‘LF’, and ‘Total Power slope’. The final set of features was as listed in Table A3 of the Appendix A.

When greedy feature selection was performed by evaluating the error metric of the MLR model, it was observed that the forward feature selection set contained 36 features. Out of these, the backward elimination algorithm removed 4 features, ‘HF’, ‘Max. Speed’, ‘Total Power’, ‘HR MAX 25’, to improve the model performance. This resulted in the final 32 optimal features for $VO_{2max}$ prediction, as shown in Table A3 of the Appendix A. While using the RF model for the above-mentioned features selection method, it was observed that in total, there were 18 features selected by the forward algorithm such as ‘Ex. Duration’, ‘R TIME 75’, ‘TimeSp25’, ‘Age’, ‘Max. Speed’, ‘BMI’, from which three were removed, which are ‘Ex. Duration’, ‘R TIME 75’ and ‘CVI’, by the backward process resulting in the final set of features as shown in Table A3 of the Appendix A.

Similarly, to the exercise data, the 30 features corresponding to the recovery data, and SVR model, the forward and backward algorithm was performed iteratively to find the best set of features. It was found that the forward feature selection process yielded 10 features which are ‘Ex. Duration’, ‘BMI’, ‘Gender’, ‘Protocol Type’, ‘Max. Speed’, ‘Age’, ‘Median NNI’, ‘Height’, ‘Weight’, ‘PNNI 50’. Whereas, during the backward elimination of features, no features were removed. The final set of features were as listed in Table 3. By performing a similar process using the MLR model, the forward algorithm selected 10 features, namely, ‘Ex. Duration’, ‘BMI’, ‘Gender’, ‘Protocol Type’, ‘Max. Speed’, ‘Age’, ‘Median NNI’, ‘Height’, ‘Weight’, ‘PNNI 50’. Whereas, the removal of none of the features resulted in improved performance. Hence, the final set of features was the same as that was given by the forward algorithm as shown in Table 3.

When this features selection method was applied to the RF model, its testing error stopped improving after the addition of 17 features.

The features were ‘Ex. Duration’, ‘Weight’, ‘Age’, ‘Gender’, ‘Std. HR’, ‘Protocol Type’, ‘Max. Speed’ and ‘DFA Slope 60’. Even here, the termination condition was met without any feature removal. A complete list of selected features is shown in Table 3.

## 6. Results

The dataset was cleaned by removing the subjects with unreliable heart rate segments which accounts for 75 instances in exercise data and 20 in recovery data. These data were further divided into training and testing sets by following the norms of 10-fold cross-validation.

The dataset was randomly split into ten partitions, out of which nine were selected for training and one for testing. In a single fold during the cross-validation process, the subjects were split into two mutually exclusive sets with 90% of the subjects used for training and 10% for testing which followed the Person Independence (PI) based training. A PI-based training was employed to ensure that the subjects that were a part of training process were not included in the testing. The advantage of 10-fold cross-validation is that there are ten such combinations of training and testing sets whose aggregate metrics take the stochastic nature of the random split into account. The mechanism of train–test split is described in the Appendix A and the corresponding RMSE across the 10 folds of cross-validation observed for an instance is tabulated in Table A4.

Thereafter, the regression models were trained using the training set and their performance was measured on the testing set. All the hyperparameters of RF (‘max. depth’, ‘n estimators’ and ‘min. samples split’) and SVR (C, ϵ and γ) were tuned using the Grid-Search algorithm in conjunction with a 5-fold cross-validation. This process was performed for each variation of the RF and SVR-based model separately and subsequent results were noted.

The base features, namely, ‘Max. Speed’, ‘Ex. Duration’, ‘Max. HR’ and metadata were used to train MLR, RF and SVR models as described in Section 4 and the results were obtained as mentioned in Table 4.

A similar study was performed using SVR by Akay et al. [54] and R reported by that study was 0.71, which was in accordance with our observation of 0.70, with similar features and model. Whereas, the RMSE of the above mentioned study was 5.48 which was slightly higher than the 5.34 value reported by this study, as shown in Table 4. To improve the performance of the above-mentioned models, several HRV features were evaluated. This resulted in 101 features for the exercise data and 30 features for the recovery data, which were used to train the previously mentioned models and the results were noted as specified in Table 5.

For the exercise data as well as recovery data, R and RMSE did not show any significant change with respect to SVR but there was slight decrement in the performance metric of MLR and RF which can be seen from the Table 5. This decrease in performance was caused by the overfitting of the model due to the presence of large number of features. Therefore, to address this concern, several feature selection methods such as correlation-based, mutual information-based and greedy forward-backward feature selections were used.

### 6.1. $VO_{2max}$ Estimation during Exercise

As it is evident from Table 6, the greedy feature selection method gave the highest R values and lowest RMSE with respect to all regression models. Additionally, it can be seen that the performance of MLR was very close to the performance of SVR with greedy feature selection; on the other hand, RF gave the least desirable results. The best values of R and RMSE were 0.74 and 4.99, respectively, which was observed using MLR in conjunction with greedy feature selection. Compared to the no-feature-selection approach, there was an improvement of 5.7% and 7.2% in the R value with greedy-based SVR and MLR, respectively. There was a reduction of 5.6% and 8.1% in the RMSE of greedy-based SVR and MLR, respectively.

**Figure 6:**
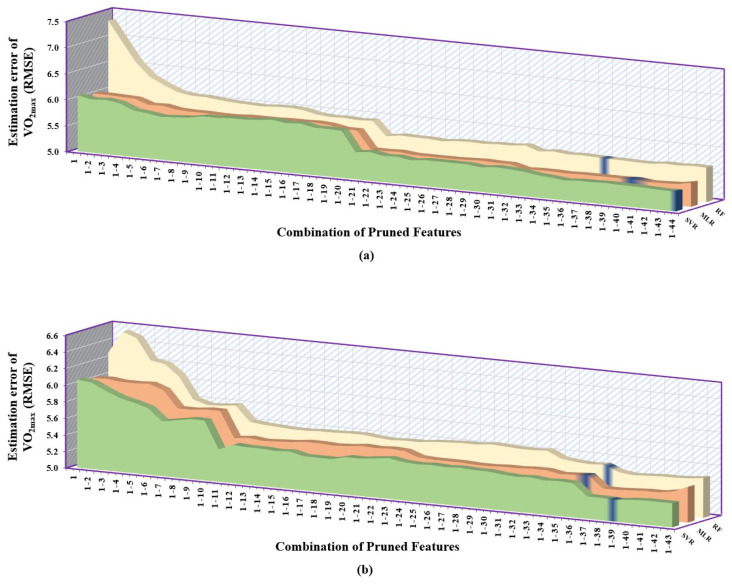
Error plot depicting the performance of regression models (MLR, RF, SVR) trained using a combination of pruned features associated with exercise data. The y–axis of each subplot shows prediction error using: (**a**) correlation–based feature selection method. (**b**) mutual information–based feature selection method. Each entry in the x–axis is represented in the form [1–N], which corresponds to the combination of 1^st^ to $N^{\mathrm{th}}$ feature set that is provided to regression models.

It can be clearly inferred from Table 6 that the correlation-based feature selection method gave the least desirable performance followed by the mutual information-based approach, and the greedy-based method provided the overall best results. When the correlation-based feature selection method was employed, SVR performed best when given with first 44 pruned features followed by MLR with first 41 pruned features and RF gave the highest RMSE consistently but only took 39 pruned features to give its best result. From the Figure 6a, we can see that SVR performed consistently better than the other two models, whereas, among MLR and RF, MLR had significantly lower error than RF.

While selecting the features using the mutual information-based method, it was observed that the SVR had the overall lowest RMSE followed by MLR and RF, as shown in Figure 6b. It can also be observed that the number of pruned features for which the models gave their best performance lied in close proximity. For SVR, it was first 39 pruned features, MLR had first 37 pruned features and RF had first 38 pruned features.

### 6.2. $VO_{2max}$ Estimation during Recovery

In contrast to the exercise data, the number of subjects with unreliable HR segments was far smaller. In total, 17 such subjects were removed, which accounts for only 1.71% of the subjects taken in this study. Similar to the exercise data, feature selection and model training were performed and the evaluation metric was noted as shown in Table 7.

As it is evident from Table 7, the greedy feature selection method gave the highest R values and lowest RMSE with respect to all but one regression models, i.e., RF. Additionally, it can be seen that the performance of MLR was very close to the performance of RF with respect to the greedy and correlation-based feature selection method.

The overall best values of R and RMSE values were 0.73 and 5.15, respectively, which was observed using SVR in conjunction with greedy feature selection. Compared to the no-feature-selection approach, there was an improvement of 2.8% and 2.1% in R and RMSE value, respectively, with greedy-based SVR. It can be inferred from the Table 7 that the performance of correlation and mutual information-based MLR and SVR were undiscernable as in the case of mutual information and greedy-based RF.

**Figure 7:**
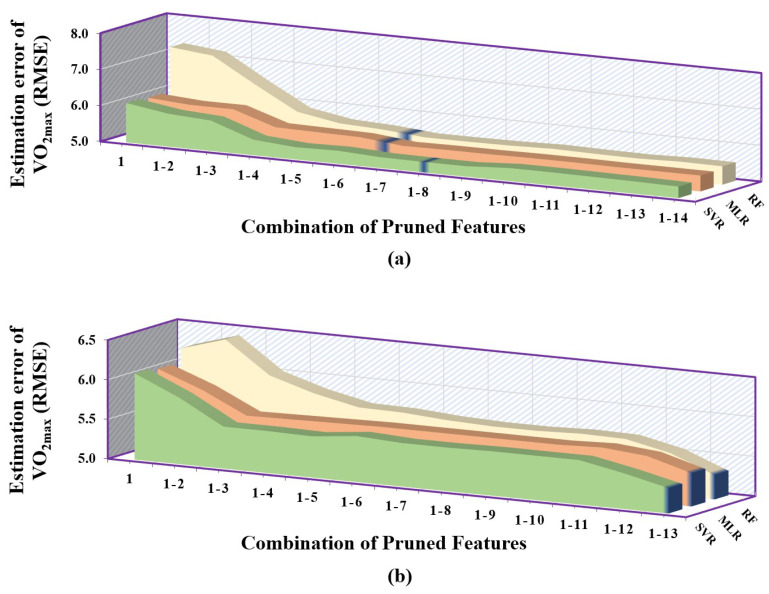
Error plot depicting the performance of regression models (MLR, RF, SVR) trained using a combination of pruned features associated with recovery data. The y–axis of each subplot shows prediction error using: (**a**) correlation–based feature selection method. (**b**) mutual information–based feature selection method. Each entry in the x–axis is represented in the form [1–N], which corresponds to the combination of 1st to *N*th feature set that is provided to regression models.

When the correlation-based feature selection method was employed, SVR performed best when given with the first eight pruned features followed by RF with the first seven pruned features and MLR gave the highest RMSE with the first seven pruned features. As it is evident from Figure 7, SVR performed consistently better than the other two models, whereas, among MLR and RF, MLR had lower error than RF with respect to pruning process. While selecting the features using the mutual information-based method, it was observed that the SVR had the overall lowest RMSE followed by RF and MLR, as shown in Figure 7b. It can also be observed that the number of pruned features for which the models gave their best performance was exactly the same. These best features contained the complete pruned feature set of 13 features.

## 7. Post-Modeling Analysis

In this section, the post-modeling analysis was performed to gauge the effect of deviant points on the model performance. The deviant points were those that deviated too far from the primary cluster of data in the feature space formed by the measured $VO_{2max}$ and Max. Speed. Based on the previous feature selection approach, it was observed that Max. Speed was not only a highly ranked feature, but was also consistently present in the final feature sets of most of the models. Its Spearman’s correlation coefficient with the measured $VO_{2max}$ was also significantly high, which indicated a monotonic relationship between the two. Therefore, the two-dimensional feature space was constructed using Max. Speed and measured $VO_{2max}$ and the k-NN-based method was applied to detect the deviant points. The deviant points, due to their divergent behavior, would compromise the generalizability of the model. The deviant points were scattered away from the main cluster formed by the reliable counterparts. Neither a discernible pattern was observed among the deviant points nor the deviant points that constituted the isolated cluster occurred in large numbers. Hence, the presence of deviant points could hamper the training process by deviating the model from learning the optimal parameters, thereby affecting the prediction outcomes of reliable measurements.

### 7.1. Case I

The model results, without addressing these deviant points, would not reflect the scenario when the models are exposed to a dataset with reliable measurements. Hence, the model performance was re-evaluated by removing these observations from the whole dataset, redoing the training, carrying out the features selection process and testing the model based on the methods as described in the previous sections. By using the k-NN-based method to remove deviant points, in total, 89 data points were detected as unreliable. MLR and SVR models along with greedy-based feature selection were chosen due to their superior performance when compared with other combinations of models and feature selection methods. In case of exercise data, greedy-based MLR performed slightly better than greedy-based SVR. For the recovery data, MLR performed better, unlike in the case of data with deviant points where SVR performed better than MLR. There was a 5.1% increment in the R-value and a significant decrease of 12.2% in RMSE of greedy-based MLR due to the removal of deviant points. Whereas, for the recovery data, the increment in R-value was found to be 7.0% and RMSE was decreased by 13.6%. The overall improvement of R and RMSE was 13.0% and 19.3%, respectively, when compared with the initial results of the same model. The removal of deviant points, both in training and testing, effectively improved the model performance in Case I.

### 7.2. Case II

The approach used in Section 7.1 cannot be used in $VO_{2max}$ estimation for a downstream application since it requires ground-truth $VO_{2max}$ to identify deviant points. Therefore, another case was included in the post-modeling analysis where the deviant points were removed only from the training and the models were exposed to outliers during evaluation which mimicked the real-world scenario. The similar steps were followed as described in Subsection refcase1. In this case, the k-NN-based method was used to remove deviant points only from the training set. On the other hand, the ratio of reliable-to-deviant points in the testing set was maintained similar to their ratio that existed in entire dataset. MLR and SVR models along with greedy-based feature selection were chosen in this case due to their superior performance. For the recovery data, SVR performed slightly better than MLR as similar in the case of data with deviant points. There was a 4.1% decrement in the R-value and increase of 4.8% in RMSE of greedy-based SVR due to the removal of deviant points only from the training set. Whereas, for the exercise data, greedy-based SVR performed slightly better than greedy-based MLR. The decrement in R-value was observed to be 1.4% and the increase in RMSE was observed to be 4.2%. The removal of deviant points, only while training the model, reduced the model performance as expected since the model parameters were not tuned to generalize for the divergent behavior of deviant points. The Case I and Case II results noted for exercise and recovery are as shown in Table 8 and Table 9, respectively.

## 8. Discussion

In this study, the feasibility of using HRV to estimate $VO_{2max}$ during exercise and recovery phase was examined with the dataset comprised of 991 measurements. By using different HRV features extracted from exercise and recovery segments separately, as mentioned in Section 3.3, three different Machine Learning-based regression models—MLR, RF and SVR—were employed in order to estimate $VO_{2max}$. A total of 101 features were extracted from the exercise phase and 30 features were extracted from HR collected during recovery. In order to avoid overfitting of the model and improve the performance of the model, pruning of features was performed using correlation-based, mutual information-based and greedy-based feature selection methods. Among all the feature selection and model combinations used in the study, greedy-based MLR yielded better results (R = 0.74 & RMSE = 4.99) with respect to exercise features and greedy-based SVR showed best results (R = 0.73 & RMSE = 5.15) with respect to recovery features. Further, to increase the robustness of the model, k-NN-based clustering was used to remove the outliers. After the removal of outliers, there was significant decrease in RMSE overall for both exercise and recovery segments. The addition of pruned HRV features extracted from both exercise as mentioned in Appendix A and recovery segments as mentioned in Table 1, Table 2 and Table 3, increased the model performance and hence, this study conforms the feasibility of using HRV, extracted from both exercise and recovery phase, as a predictor variable to assess $VO_{2max}$. Additionally, a greater number of HRV features was extracted from exercise HR when compared with recovery HR since the duration of recovery phase was only 3 min. Despite the limited number of features, the evaluation metric of model using recovery HRV differed from exercise HRV by only 0.02. HR was obtained from athletes performing GET protocol on a treadmill and hence, the data recorded were more susceptible to motion artifacts. As elaborated in Section 3.2, a total of 72 data points from the exercise segment and 17 data points from recovery, which had irrelevant HR measurements, were removed to avoid bias in HRV interpretation. With respect to the higher incidence of extracting HRV information from reliable HR segments and similarity between the evaluation metrics, HRV from recovery phase could be a alternate potential candidate in the assessment of $VO_{2max}$.

Studies have established the fact the recovery rate is directly proportional to the cardiorespiratory fitness of the athletes (i.e.,) if the recovery rate of the individual is faster or quicker, the vagal activity is stimulated post exercise, as measured by HRV, the individual would be more fitter as measured by $VO_{2max}$. In our study, two of the HRV features that were consistently ranked superior with high relevance with $VO_{2max}$ during the recovery were DFA slope estimate $\alpha_{1}$ and PNNI 50. Moreover, a study [19] that examined HRV features, illustrated that PNNI 50 derived from exercise HR showed a significant impact on the estimation of $VO_{2max}$. Thus, it is clear that more HRV-related experimental studies in both exercise and recovery phase should be conducted to validate the physiological relevance between HRV and $VO_{2max}$.

Therefore, this study proposes the utility of HRV in the assessment of $VO_{2max}$ of athletes of large sample size with wide age range distribution. Our study is one of a kind as it examines HRV, extracted from exercise and recovery phase, to estimate $VO_{2max}$ on a larger population of athletes. Additionally, this study methodology supports the use of wearable HR monitors and hence, they can be used in place of the conventional obtrusive setup for $VO_{2max}$ assessment. Furthermore, due to the unobtrusive nature of wearable HR monitors, the $VO_{2max}$ assessment of athletes can be conveniently performed without affecting their functional capability at maximal intensities, unlike the conventional setup.

## 9. Conclusions

In this work, we propose the utility of HRV extracted from exercise and recovery HR segments in assessing the $VO_{2max}$ of a large number of athletes. We employed regression models to estimate $VO_{2max}$ from the HRV feature set. We incorporated feature selection methods with the regression models in order to avoid the overfitting of the model. Eventually, pruned HRV features which had high relevance with the model estimate, presented a significant impact in the estimation of $VO_{2max}$ by showing improved model performance. Furthermore, we applied k-NN on the data points of exercise and recovery, which resulted in an increase in model’s accuracy and reduction in RMSE, thereby optimizing the model performance. With respect to the reliability of HR measurements and optimized model performance with lesser pruned features, the use of HRV from recovery phase could be a potential candidate in assessing the $VO_{2max}$ of individuals. Therefore, this work contributes to the utility of wearable HR monitors for the athletes by validating the use of HRV information, extracted from exercise and recovery phase, for the assessment of $VO_{2max}$.

In our future work, we intend to explore the potential of each HRV derived from recovery phase, by examining the recovery data of sufficient window length as the scope of the current study was to examine the HRV derived from recovery window of 3 min. Further, we would like to explore the importance of gaining a greater amount of information from HRV using feature stacking for improving the model performance. Eventually, we intend to demonstrate the utility of HRV-based $VO_{2max}$ assessment of athletes using wearable HR monitors.

## Figures and Tables

**Table 1:** Pruned features based on Correlation during Recovery.

Category	Pruned Features	Description
METADATA	**Age**	Physical Attributes
	**Height**	
	**BMI**	
	**Gender**	
	**Protocol Type**	Information on whether protocol is “step” or “incremental”
HR -HRV FEATURES	**Std. HR**	Standard Deviation of HR
	**Min HR**	Lowest Heart Rate value
	**NNI 50**	Number of successive NNI intervals that differ by more than 50 ms
	**PNNI 20**	Percentage of successive NNI intervals that differ by more than 20 ms
	**Ins DFA Slope 120**	Average of Instantaneous Slope of DFA values between 0–120 s
	**Ins DFA Slope 180**	Average of Instantaneous Slope of DFA values between 0–180 s
	**DFA Slope 120**	Slope of DFA values between 0–120 s where DFA describes long term fluctuations in NNI series
	**DFA Slope 180**	Slope of DFA values between 0–180 s
TIME BASED FEATURES	**Ex. Duration**	Effective Time spent on Treadmill in seconds

**Table 2:** Pruned features based on Mutual Information during Recovery.

Category	Pruned Features	Description
METADATA	**Age**	Physical Attributes
	**BMI**	
	**Gender**	
	**Protocol Type**	Information on whether protocol is “step” or “incremental”
HR - HRV FEATURES	**Std. HR**	Standard Deviation of HR values
	**Min HR**	Lowest Heart Rate value
	**SDSD**	Standard Deviation of Successive Difference of NNI intervals
	**NNI 20**	Number of successive NNI intervals that differ by more than 20 ms
	**NNI 50**	Number of successive NNI intervals that differ by more than 50 ms
	**Ins DFA Slope 180**	Average of Instantaneous Slope of DFA values between 0–180 s where DFA describes long term fluctuation in NNI series
	**DFA Slope 60**	Slope of DFA values between 0–60 s
	**DFA Slope 180**	Slope of DFA values between 0–180 s
TIME BASED FEATURES	**Max. Speed**	Maximum speed reached

**Table 3:** Best features based on Greedy feature selection during Recovery.

Category	Greedy Feature Selection
MLR	Age, Gender, Height, Weight, BMI, Protocol type, Median NNI, PNNI 50, Max. Speed, Ex. Duration
RF	Age, Gender, Weight, Height, BMI, Protocol type, Max. HR, Std. HR, Range NNI, SDSD, NNI 20, PNNI 20, DFA Slope 60, Ins DFA Slope 180, CVNNI, Ex. Duration, Max. Speed
SVR	Age, Gender, Weight, BMI, Protocol type, Range NNI, NNI 20, Ex. Duration, Max. Speed

**Table 4:** Performance Metrics of models with Base Features.

Model Description	Features—Max Speed, Exercise Duration Max. HR and Metadata	
	R	RMSE
MLR	0.69	5.43
RF	0.67	5.58
SVR	0.70	5.34
Akay et al. [54] (SVR)	0.71	5.48

**Table 5:** Performance Metrics of models with all the features.

Model Description	During Exercise		During Recovery	
	R	RMSE	R	RMSE
MLR	0.67	5.71	0.68	5.57
RF	0.67	5.58	0.69	5.40
SVR	0.71	5.28	0.71	5.26

**Table 6:** Performance Metrics of Models for Exercise Data.

Feature Selection Method	MLR		RF		SVR	
	R	RMSE	R	RMSE	R	RMSE
Correlation	0.69	5.40	0.67	5.61	0.71	5.31
Mutual Information	0.71	5.31	0.69	5.44	0.72	5.23
Greedy	0.74	4.99	0.71	5.26	0.74	5.04

**Table 7:** Performance Metrics of Models for Recovery Data.

Feature Selection Method	MLR		RF		SVR	
	R	RMSE	R	RMSE	R	RMSE
Correlation	0.69	5.43	0.69	5.39	0.71	5.28
Mutual Information	0.69	5.42	0.71	5.29	0.71	5.30
Greedy	0.71	5.29	0.71	5.28	0.73	5.15

**Table 8:** Models performance metrics for Case I and II during exercise.

Model Description	Performance Metrics for Exercise			
	Case I		Case II	
	R	RMSE	R	RMSE
Greedy-SVR	0.78	4.42	0.73	5.20
Greedy-MLR	0.78	4.38	0.72	5.26

**Table 9:** Models performance metrics for Case I and II during recovery.

Model Description	Performance Metrics for Recovery			
	Case I		Case II	
	R	RMSE	R	RMSE
Greedy-SVR	0.75	4.62	0.70	5.40
Greedy-MLR	0.76	5.26	0.70	5.42

## Data Availability

The dataset Treadmill Maximal Exercise Tests from the Exercise Physiology and Human Performance Lab of the University of Malaga is available on the website https://physionet.org/content/treadmill-exercise-cardioresp/1.0.1/ (accessed on 31 October 2022).

## Abbreviations

The following abbreviations are used in this manuscript:

HR	Heart Rate
HRV	Heart Rate Variability
$VO_{2max}$	Maximal oxygen uptake
CPET	Cardio Pulmonary Exercise Test
CRF	Cardio Respiratory Fitness
ML	Machine Learning
MLR	Multiple Linear Regression
RF	Random Forest
SVR	Support vector Regression

## Appendix A

**Table A1:** Correlation–based Pruned Features for exercise data.

Category	Pruned Features	Description
METADATA	**Age**	Physical Attributes
	**BMI**	
	**Gender**	
	**Protocol Type**	Information on whether protocol is “step” or “incremental”
HR-HRV FEATURES	**Sum TRIMP**	Cumulative sum of TRaining IMPulse values calculated using Bannister Equation
	**Min HR**	Lowest Heart Rate value where Min HR = minimum of total HR
	**Std. HR**	Standard Deviation of HR values
	**NNI 20**	Number of successive NNI intervals that differ by more than 20 ms
	**NNI 50**	Number of successive NNI intervals that differ by more than 50 ms
	**Range NNI**	Difference between Maximum NNI and Minimum NNI
	**VLF**	Absolute power of the very-low-frequency band (0.0033–0.04 Hz)
	**LF**	Absolute power of the low-frequency band (0.04–0.15 Hz)
	**HF**	Absolute power of the high-frequency band (0.15–0.4 Hz)
	**LF_nu**	LF/(LF + HF) LF = Absolute power of low frequency (0.04–0.15 Hz) where HF = Absolute power of high frequency (0.15–0.4 Hz)
	**DFA at maximum HR**	Detrended fluctuation analysis, which describes long-term fluctuations at maximum HR
	**Sample Entropy**	Measure of regularity and complexity of a NN series
	**Triangular ind**	Integral of the density of the RR interval histogram divided by its height
	**Mean HR Slope**	Slope of Mean HR computed for every 2 min window where Mean HR = mean of total HR
	**NNI 20 Slope**	Slope of NNI 20 computed for every 2 min window where NNI 20 = Number of successive NNI intervals that differ by more than 20 ms
	**PNNI 50 Slope**	Slope of PNNI 50 computed for every 2 min window where PNNI 50 = Percentage of number of successive NNI intervals that differ by more than 50 ms
	**LF Slope**	Slope of LF power values computed for every 2 min window
	**HF Slope**	Slope of HF power values computed for every 2 min window
	**DFA Slope**	Slope of DFA values computed for every 2 min window
	**SD2 SD1 Slope**	Slope of SD2 SD1 computed for every 2 min window SD1—standard deviation of projection of the Poincaré plot on the line perpendicular to the line of identity SD2—standard deviation of projection of the Poincaré plot on the line on the line of identity
	**Triangular ind Slope**	Slope of Triangular indexes for every 2 min window
	**CVNNI Slope**	Slope of Co-Variance of NNI intervals where CVNNI = Ratio of standard deviation of NNI and mean NNI
	**SlopeSp25**	Slope of HR and Time segments corresponding to 0–25% maximum speed
	**SlopeSp50**	Slope of HR and Time segments corresponding to 25–50% maximum speed
	**SlopeSp75**	Slope of HR and Time segments corresponding to 50–75% maximum speed
	**SlopeSp100**	Slope of HR and Time segments corresponding to 75–100% maximum speed
TIME BASED FEATURES	**Exercise Duration**	Effective Time spent on Treadmill in seconds
	**RT50**	Correlation between speed and HR segments corresponding to 25–50% total time
	**RT75**	Correlation between speed and HR segments corresponding to 60–75% total time
	**RSp75**	Correlation between speed and HR segments corresponding to 0–75 % maximum speed
	**TimeHR25**	Time at which their HR was 25% max HR
	**TimeHR50**	Time at which their HR was 50% max HR
	**TimeHR75**	Time at which their HR was 75% max HR
	**DurationHR60**	Total time spent in zone 50–60% of max HR
	**DurationHR70**	Total time spent in zone 60–70% of max HR
	**DurationHR80**	Total time spent in zone 70–80% of max HR
	**DurationHR90**	Total time spent in zone 80–90% of max HR
	**DurationHR100**	Total time spent in zone 90–100% of max HR

**Table A2:** Mutual Information–based Pruned Features for exercise data.

Category	Pruned Features	Description
METADATA	**Age**	Physical Attributes
	**BMI**	
	**Gender**	
	**Protocol Type**	Information on whether protocol is “step” or “incremental”
HR-HRV FEATURES	**Sum TRIMP**	Cumulative sum of TRaining IMPulse values calculated using Bannister Equation
	**Min HR**	Lowest Heart Rate value
	**25% HR max**	25% of maximum HR value
	**PNNI 20**	Percentage of number of successive NNI intervals that differ by more than 20 ms
	**PNNI 50**	Percentage of number of successive NNI intervals that differ by more than 50 ms
	**VLF**	Absolute power of the very low frequency band (0.0033–0.04 Hz)
	**LF**	Absolute power of the low frequency band (0.04–0.15 Hz)
	**HF**	Absolute power of the high frequency band (0.15–0.4 Hz)
	**LF/HF**	Ratio of LF–HF power
	**DFA at maximum HR**	Detrended fluctuation analysis, which describes long-term fluctuations at maximum HR
	**Triangular ind**	Integral of the density of the RR interval histogram divided by its height
	**CVSD**	Coefficient of variation of successive differences equal to the rmssd divided by Mean NNI
	**Std. HR Slope**	Slope of standard deviation of HR computed for every 2 min window
	**Mean HR Slope**	Slope of Mean HR computed for every 2 min window
	**NNI 20 Slope**	Slope of NNI 20 computed for every 2 min window
	**SD2 SD1 Slope**	Slope of SD2 SD1 computed for every 2 min window SD1—standard deviation of projection of the Poincaré plot on the line perpendicular to the line of identity SD2—standard deviation of projection of the Poincaré plot on the line on the line of identity
	**SDSD Slope**	Slope of standard deviation of successive difference of NNI computed for every 2 min window
	**PNNI 50 Slope**	Slope of PNNI 50 computed for every 2 min window
	**HF Slope**	Slope of HF power values computed for every 2 min window
	**Total Power Slope**	Slope of Total power computed for every 2 min window, Total power = LF + HF
	**DFA Slope**	Slope of DFA values computed for every 2 min window
	**Triangular ind Slope**	Slope of Triangular index values computed for every 2 min window
	**Sample Entropy Slope**	Slope of sample entropy values computed for every 2 min window
	**SlopeSp25**	Slope of HR values between 0–25% of maximum speed reached
	**SlopeSp50**	Slope of HR values between 25–50% of maximum speed reached
	**SlopeSp75**	Slope of HR values between 50–75% of maximum speed reached
	**SlopeSp100**	Slope of HR values between 75–100% of maximum speed reached
TIME BASED FEATURES	**RT75**	Correlation between speed and HR segments corresponding to 50–75 % total time
	**RT100**	Correlation between speed and HR segments corresponding to 75–100% total time
	**R Total**	Correlation of speed and HR for total duration
	**Max. Speed**	Maximum speed reached
	**TimeHR25**	Time at which their HR was 25% max HR
	**TimeHR75**	Time at which their HR was 75% max HR
	**DurationHR70**	Total time spent in zone 60–70% of max HR
	**DurationHR80**	Total time spent in zone 70–80% of max HR
	**DurationHR90**	Total time spent in zone 80–90% of max HR
	**DurationHR100**	Total time spent in zone 90–100% of max HR

**Table A3:** Best Features selected based on Greedy-based Feature selection on exercise segments.

Models	Greedy Feature Selection
Multiple Linear Regression	Age, Gender, Height, Weight, BMI, Protocol Type, Sum TRIMP, Min HR, PNNI 20, PNNI 50, SD2, Mean NNI Slope, Std. HR Slope, Range NNI Slope, NNI 50 Slope, PNNI 50 Slope, LF Slope, SD1 Slope, SD2 Slope, SlopeSp25, SlopeSp100, RSp25, RSp50, RSp100, TimeSp50, TimeSp75, TimeSp100, RT50, RT75, DurationHR60, DurationHR70, DurationHR100, Ex. Duration
Random Forest	Age, Gender, Height, BMI, Protocol Type, LF/HF, HF_nu, DFA at maximum HR, PNNI 20 Slope, LF Slope, Triangular ind Slope, TimeSp25, RT75, DurationHR90, Max. Speed
Support Vector Machine	Age, Gender, Weight, BMI, Protocol Type, Sum TRIMP, Mean TRIMP, NNI 20, VLF, Min HR Slope, Std. HR Slope, Range NNI Slope, NNI 20 Slope, PNNI 20 Slope, LF Slope, SD2 Slope, Modified CSI Slope, RSpAvg, RSp75, TimeSp50, TimeSp75, RT75, TimeHR25, TimeHR50, DurationHR70, Max. Speed, Ex. Duration

**Table A4:** Tabulation of observed RMSE values across 10 cross-validation folds.

CVs	Testing Set	RMSE
CV1	12, 13, 26, ……..	5.351
CV2	5, 21, 37, ……..	5.039
CV3	8, 19, 33, ……..	5.187
CV4	6, 24, 48, ……..	5.046
CV5	7, 11, 15, ……..	4.967
CV6	10, 43, 74, ……..	5.737
CV7	2, 17, 18, ……..	5.541
CV8	3, 4, 16, ……..	5.622
CV9	14, 20, 28, ……..	5.318
CV10	7, 9, 15, ……..	5.218

The mean and standard deviation of RMSE computed across 10 cross-validation folds were 5.303 and 0.262, respectively. To help with the reproducibility of the results, the data were split into training and testing using the sklearn.model_selection.Kfold module with random_state set to a seed value of 5.

This K-Folds cross-validator module provided indices to split the data into train/test sets and split the dataset into k consecutive folds. Each fold was then used once as validation while the k–1 remaining folds were used to train the model. Using the fixed seed for the random state ensured that indices obtained using random shuffles would be replicable.
